# Single-cell transcriptome profiling reveals enriched memory T-cell subpopulations in hypertension

**DOI:** 10.3389/fcell.2023.1132040

**Published:** 2023-03-16

**Authors:** Xiaoqi Wang, Xiaobin Wu, Pei Zhang, Yuan Zhou, Jun Cai, Ling Jin

**Affiliations:** ^1^ Hypertension Center, Fuwai Hospital, Chinese Academy of Medical Sciences and Peking Union Medical College, State Key Laboratory of Cardiovascular Disease, National Center for Cardiovascular Diseases, Beijing, China; ^2^ Department of Biomedical Informatics, School of Basic Medical Sciences, Ministry of Education Key Laboratory of Molecular Cardiovascular Sciences, Peking University, Beijing, China; ^3^ Department of Hypertension, Zhengzhou Central Hospital Affiliated to Zhengzhou University, Henan, China; ^4^ Center of Basic Medical Research, Institute of Medical Innovation and Research, Peking University Third Hospital, Beijing, China

**Keywords:** single-cell transcriptome profiling, memory T cells, hypertension, inflammation, CD8 effector memory T cells

## Abstract

**Introduction:** The adaptive immune response mediated by T cells plays a vital role in the initiation and maintenance of blood pressure (BP) elevation. Memory T cells, which are antigen-specific T cells, can respond specifically to repeated hypertensive stimuli. Although the roles of memory T cells in animal models are well studied, their maintenance and functions in hypertensive patients are poorly understood.

**Method:** Here, we focused on the circulating memory T cells of hypertensive patients. By using single-cell RNA sequencing technology, subsets of memory T cells were identified. Differentially expressed genes (DEGs) and functional pathways were explored for related biological functions in each population of memory T cells.

**Result and Discussion:** Our study identified four subsets of memory T cells in the blood of hypertensive patients, with CD8 effector memory T (TEM) cells accounting for more cells and demonstrating more biological functions than CD4 TEM cells. CD8 TEM cells were further analyzed using single-cell RNA sequencing technology, and subpopulation 1 was demonstrated to contribute to BP elevation. The key marker genes CKS2, PLIN2, and CNBP were identified and validated by mass-spectrum flow cytometry. Our data suggest that CD8 TEM cells as well as the marker genes could be preventive targets for patients with hypertensive cardiovascular disease.

## Introduction

Hypertension continues to be the leading cause of death and disability worldwide (GBD 2017 Risk Factor Collaborators, 2018). Over the years, it has been widely acknowledged that low-grade inflammation is an important mediator for the initiation and maintenance of blood pressure (BP) elevation (Norlander et al., 2018). The adaptive immune response mediated by T cells plays a vital role in BP regulation by increasing the production of chemokines and cytokines and the release of reactive oxygen species (ROS) (Caillon et al., 2019; Drummond et al., 2019). The blood vessels, kidneys, heart, and autonomic nervous system have been implicated in the control of T cells. On the basis of surface markers, T cells are classified as either CD4-positive or CD8-positive after leaving the thymus. Upon encountering antigens, naive CD4 T cells differentiate into effector Th1, Th2, Th17, or regulatory T (Treg) cells, whereas naive CD8 T cells mature into cytotoxic T cells that secrete the cytokines IFN-γ, TNF-α, perforin, and granzyme B (Zhang and Bevan,2011). The majority of effector T cells die upon clearance of infections; however, a small number of them persist in the host and are known as memory T cells. Staining for CD45RA and CCR7 can be used to categorize T cells into naïve T cells (CD45RA^+^/CCR7^+^), TEM cells (CD45RA^−^/CCR7^−^), central memory T (TCM) cells (CD45RA^−^/CCR7^+^), and effector memory cells re-expressing CD45RA (TEMRA) cells (CD45RA^+^/CCR7^−^) (Willinger et al., 2005). As T-cells transition from naïve to effector to memory cells, their overall gene expression profiles change, resulting in phenotypic and functional variations among the different populations (Kalia and Sarkar, 2018; Swain et al., 2021).

Hypertensive stimuli, such as sleep apnea, repeated episodes of dietary indiscretion, and emotional stress, are intermittent and reoccurring. These repeated stimulations result in the existence and response of specific memory T cells in hypertensive patients, which can be viewed as “immunological memory” of hypertension. It has been consistently observed that memory T cells increase in number in the blood, vasculature, and kidneys of hypertensive mice (Itani et al., 2016a). However, the maintenance and functions of memory T cells in hypertensive patients are poorly understood. In our study, we explored the roles of peripheral CD4 and CD8 memory T cells and the immunological memory features in hypertensive patients at the single-cell level and identified key genes for these immune cells.

## Methods

### Recruitment of human participants

Five male normotensive control individuals (systolic blood pressure (SBP) ≤ 130 mmHg and/or diastolic blood pressure (DBP) ≤ 85 mmHg) and five male hypertensive patients (SBP > 130 mmHg or DBP > 80 mmHg) were strictly enrolled into the current study. All participants were aged between 40 and 55 years. Those with hyperlipidemia, hepatobiliary disorders, coronary heart disease, diabetes mellitus, heart failure, renal failure, tumor, smoking, drinking, stroke, and peripheral artery disease were excluded, and none of the patients was under antihypertensive treatment. BP was measured in a sitting position by nurses or physicians. Three readings were recorded at 5-min intervals using a random-zero mercury column sphygmomanometer, and the average was taken as the final measurement. Information on the participants is provided in Table 1. The study was conducted under a protocol approved by the Ethics Committee of Fuwai Hospital.

**TABLE 1:** Basic characteristics of normotensive controls and hypertensive patients for single-cell RNA sequencing.

	CTN (n = 5)	HTN (n = 5)	*p*-value
Age, y	47.00 ± 3.07	46.20 ± 1.43	0.865
Smoke, n (%)	0	0	
Men, n (%)	100	100	1.000
SBP, mmHg	117.20 ± 6.09	154.80 ± 6.62	0.0003
DBP, mmHg	77.20 ± 3.40	102.80 ± 5.08	0.0014
BMI, kg/m^2^	24.66 ± 0.71	25.68 ± 1.30	0.468
TG, mg/dL	1.15 ± 0.10	1.27 ± 0.14	0.163
HDL-C, mg/dL	1.17 ± 0.03	1.30 ± 0.13	0.466
LDL-C, mg/dL	2.59 ± 0.24	2.48 ± 0.10	0.628
FBS, mg/dL	4.32 ± 0.33	4.53 ± 0.32	0.730
ALT	16.00 ± 2.68	21.70 ± 3.28	0.288
AST	19.40 ± 1.72	21.90 ± 2.72	0.566
uric acid	364.20 ± 31.66	409.90 ± 22.76	0.351

SBP, systolic blood pressure; DBP, diastolic blood pressure; TG, triglyceride; HDL-C, high-density lipoprotein cholesterol; LDL-C, low-density lipoprotein cholesterol; FBS, fasting blood sugar; ALT, alanine transaminase; AST, aspartate transferase.

*p*-values were calculated using Student’s t-test.

### Single-cell sample preparation and single-cell RNA sequencing

Blood samples freshly collected from each participant were immediately prepared to isolate the peripheral blood mononuclear cells (PBMCs) by using the Ficoll density gradient method. Isolation of CD4^+^ and CD8^+^ memory T cells was performed by the MagniSort™ Human CD8 Memory T cell Enrichment Kit and MagniSort™ Human CD4 Memory T cell Enrichment Kit (Thermo, United States), respectively. In summary, the single-cell suspension of lymphocytes was labeled with a cocktail of biotinylated antibodies followed by streptavidin-coated magnetic beads. When cells are placed in the MagniSort^TM^ magnet, the undesired cells will be held in place by the magnetic field, while the desired memory T cells remain untouched and free in solution so that they can be isolated by decanting.

The single-cell RNA sequencing service was provided by the State Key Laboratory of Cardiovascular Disease in Fuwai Hospital, using the ICELL8 Single-Cell System. In general, the cell suspension stained with a mixture of Hoechst 33342 and propidium iodide was dispensed into the microchip nanowells. The dispensed cells were imaged, and single live cells were selected for cDNA preparation. A volume of 1 ng of purified cDNA was applied to generate a sequencing library using the Nextera XT DNA sample preparation kit (FC-131–1024, Illumina). Libraries were sequenced on the NextSeq 500 sequencer (Illumina).

### Data quality control and cell-type annotation

The per-cell expression matrix is publicly available in Figshare at https://doi.org/10.6084/m9.figshare.21762584.v1.The raw read data are available in the Genome Sequence Archive database (GSA, https://ngdc.cncb.ac.cn/gsa-human/) of the National Genomics Data Center, China, with accession no. HRA003850. We performed a quality control step for the expression data to accurately annotate the cell types. Cells with less than 200 genes or with >20% of reads mapping to mitochondrial genes were removed. The Seurat 4.0 R (Hao et al., 2021) package was used to perform cell-type annotation based on the reference mapping approach. The expression data were first normalized using the *SCTransform* function, and the cell-type labels were transferred from the reference to the query using the *FindTransferAnchors* function and *MapQuery* function. The uniform manifold approximation and projection (UMAP) was used to visualize the mapping results.

### Functional enrichment analysis

To explore the cell-type-specific functional changes between healthy and hypertensive states, we performed a gene enrichment analysis to screen the Gene Ontology (GO) functional terms, Kyoto Encyclopedia of Genes and Genomes (KEGG) pathways, and WikiPathways in both CD4^+^ memory T cells and CD8^+^ memory T cells. To achieve this, we first identified differentially expressed genes (DEGs) between two states using the *FindMarkers* function in the Seurat 4.0 R package. The Wilcoxon rank-sum test was applied to calculate the *p*-value for all genes, and the Benjamini–Hochberg method was used for *p*-value correction. Genes with an absolute value of log2-fold change > 0.25 and FDR < 0.05 were identified as the DEGs and were further submitted to g:Profiler (Raudvere et al., 2019) to investigate the significantly overrepresented GO terms, KEGG pathways, and WikiPathways.

### Cell–cell communication analysis

CellChat (Jin et al., 2021) is an R-based analysis tool to infer cell–cell communication networks. The expression profile of all cell types was used as the input file to construct the communication network. We calculated the significant ligand–receptor pairs (LRs) between any two cell types in both healthy and hypertensive states. The significant LRs were then applied to construct the cell–cell communication network.

### Trajectory analysis

To investigate the cell lineage of CD8^+^ memory T (TM) cells, the cell embeddings and cluster labels of UMAP were used to reconstruct the cell trajectory using the Slingshot R package (Street et al., 2018). The Slingshot R package learns cluster relationships in an unsupervised manner and constructs a curve for cell lineage. We used the *getLineages* function to identify the lineage structure by constructing a minimum spanning tree on the unsupervised clusters.

### Flow cytometry

For cell staining, the live cells from PBMCs were stained with Zombie NIR, followed by a 40-min incubation of surface antibodies of CD3, CD4, CD8, CD45RA, and CCR7 at 4°C. Flow cytometric analysis was performed on the full-spectrum Cytek Aurora. The details of all antibodies used are listed in Supplementary Table S1. The gating strategies for different cell sub-populations are illustrated in Supplementary Figure S1.

### CyTOF staining and data acquisition

For mass cytometry analysis, purified antibodies were obtained from BioLegend, Abcam, and BD Biosciences. Antibody labeling with the indicated metal tag was performed using the Maxpar antibody labeling kit (Fluidigm). The concentration of mass-tagged antibodies was assessed using a NanoDrop. Conjugated antibodies were titrated for optimal concentration before use. The CyTOF experiments were performed by PLTTECH (Hangzhou, China). Before each batch of samples was loaded, the instrument adjusted the signal strength of each channel according to the same bead signal (140Ce, 151Eu, 153Eu, 165Ho, and 175Lu). All samples were standardized to avoid batch effect before analysis. Data on each sample were debarcoded from raw data using a doublet-filtering scheme (Zunder et al., 2015) with unique mass-tagged barcodes. Each .fcs file generated from different batches was normalized through the bead normalization method (Finck et al., 2013). Cytobank software was applied to manually exclude debris, dead cells, and doublets, as well as to analyze live and single immune cells and annotate target cell populations.

### Statistical analysis

All validation experiment results are expressed as means ± SEM. *p*-values were calculated by Student’s t-test or the Mann–Whitney U-test; * indicates *p* < 0.05, ** indicates *p* < 0.01, and *** indicates *p* < 0.001. *p* < 0.05 was considered statistically significant.

## Results

### Single-cell RNA sequencing identifies diverse T-cell populations among memory T cells

**FIGURE 1:**
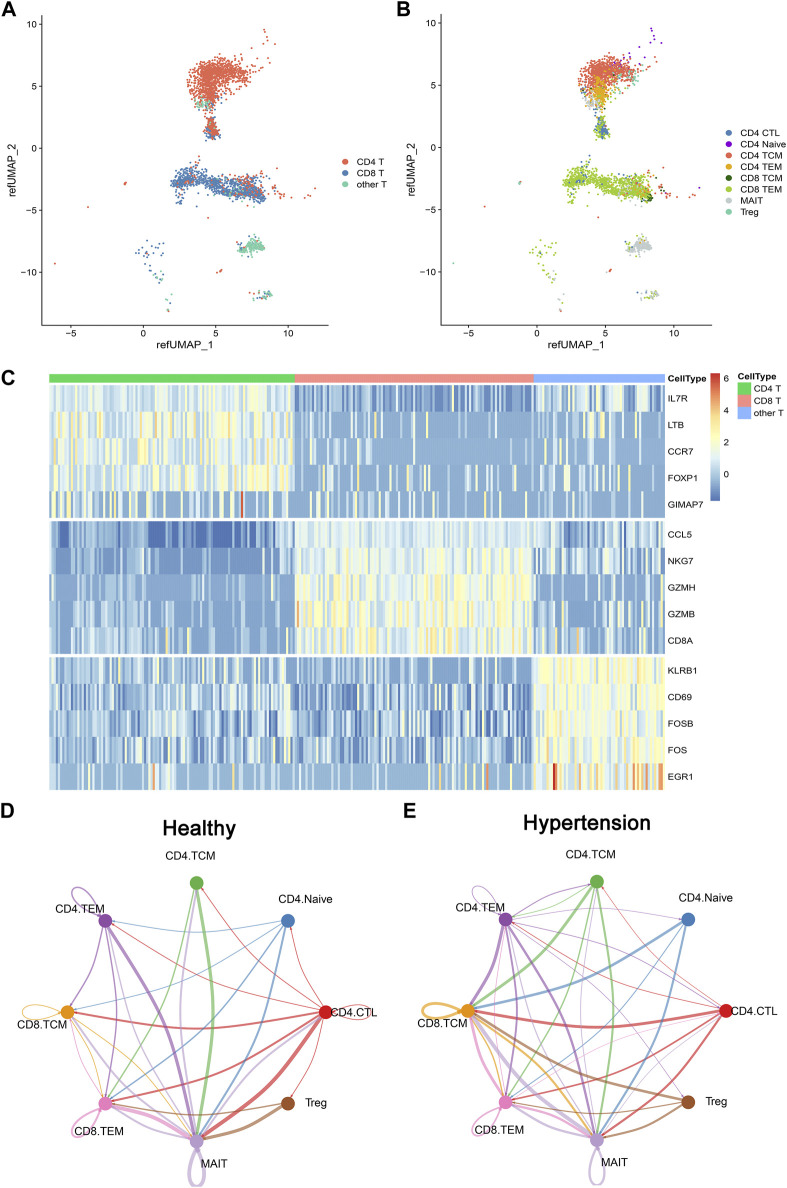
Overview of T cells from 10 integrated samples. **(A)** Uniform manifold approximation and projection (UMAP) visualization of T cells colored by three cell types. **(B)** UMAP visualization colored by eight T-cell subtypes. **(C)** Heatmap shows the expression of marker genes across CD4 T cell, CD8 T cell, and other T cells. **(D)** CellChat analysis of the communications between memory T-cell populations in healthy donors. The number of significant ligand–receptor pairs between any pair of two cell populations. The edge width is proportional to the indicated number of ligand–receptor pairs. **(E)** CellChat analysis of the communications between memory T-cell populations in hypertensive patients.

To investigate the composition of memory T-cell populations in patients with hypertension, five hypertensive patients were included along with five matched controls (Table 1). CD4^+^ and CD8^+^ memory T cells were sorted and then subjected to single-cell RNA sequencing (scRNA-seq). The basic per-sample statistics of the scRNA-seq results are available in Supplementary Table S2. After quality control of the sequencing data, 3,484 cells were retained for further analysis. We used the Seurat R package to automatically annotate cell types or subtypes. Three major cell types covering eight T-cell populations (subtypes) were identified: CD4 T cells (cytotoxic CD4 [CD4 CTL], CD4 Naïve, CD4 TCM, and CD4 TEM), CD8 T (CD8 TCM and CD8 TEM), and other T cells (mucosa-associated invariant T [MAIT] and Treg) (Figures 1A,B). Each cell type had a corresponding cell marker gene expression pattern (Figure 1C).To investigate the cell–cell communication network in both healthy and hypertensive states, CellChat analysis was run on the scRNAsequencing (scRNA-seq) dataset, which included CD4 TCM, CD4 TEM, CD8 TCM, and CD8 TEM groups, as well as several other cell types, such as CD4 Naïve, CD4 CTL, MAIT, and Treg cells. We found that the interaction of various immune cell types was enhanced in a hypertensive state compared to a healthy state (Figures 1D,E), which suggests the potential role of these cell types in the development of hypertension.

### The relative abundance of TEM cells is significantly altered in patients with hypertension

**FIGURE 2:**
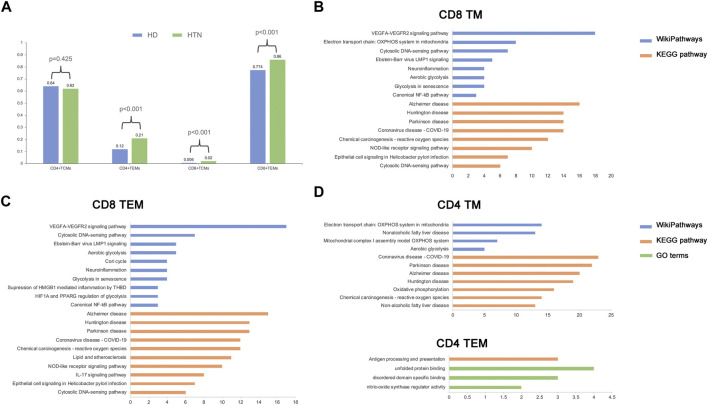
Alteration of cell frequency and activated pathways during hypertension. **(A)** Single-cell RNA sequencing assay comparing cell composition between healthy and hypertensive participants. **(B–D)** Top enriched pathways of differentially expressed genes (DEGs) in CD8 TM **(B)**, CD8 TEM **(C)**, CD4 TM, and CD4 TEM **(D)**.

The relative proportions of TCM and TEM cells in the blood vary in the CD4 and CD8 compartments in healthy human adults; the TCM type is predominant in CD4 T cells, and the TEM type is predominant in CD8 T cells (Sallusto et al., 2004). We observed similar results in our study (Figure 2A). The percentage of CD4 TCM cells among all CD4 memory T cells was comparable between the peripheral blood of healthy donors and patients with hypertension according to Fisher’s exact test [percentage: hypertensive group (HTN) versus normotensive control group (NC), 0.62 vs. 0.64, respectively; *p* = 0.425]. The memory T-cell populations that were markedly elevated in the peripheral blood of patients with hypertension were CD4 TEM cells [percentage: HTN vs. NC, 0.21 vs. 0.12, respectively; *p* = 2.9*10^−7^], CD8 TEM cells [percentage: HTN vs. NC, 0.86 vs. 0.74, respectively; *p* = 0], and CD8 TCM cells [percentage: HTN vs. NC, 0.02 vs. 0.006, respectively; *p* = 0.00027] (Figure 2A). However, CD8 TCM cells were extremely rare in human peripheral blood. Taken together, these results indicate that the composition of the memory T-cell population in hypertensive patients is altered, with an increased relative abundance of TEM cells.

### Several key pathways are activated in memory T cells in hypertension disease

To explore the biological functions related to each memory T-cell population, the differentially expressed genes (DEGs) and functional pathways were determined following the abovementioned method (see Methods). In CD8 memory T cells, several pathways associated with mitochondrial oxidative metabolism, vascular endothelium, and immune inflammation were activated during hypertension; examples include VEGFA-VEGFR2 signaling, aerobic glycolysis, and the canonical NF-kB pathway (Figure 2B). A similar result was found in CD8 TEM cells (Figure 2C). In CD4 memory T cells, in addition to mitochondrial metabolism pathways, we also found pathways related to non-alcoholic fatty liver disease, which has been shown to be associated with the development of hypertension. However, only a small number of pathways were enriched in CD4 TEM cells, which suggests that these cells underwent relatively few functional changes in hypertension. Moreover, we found that several Gene Ontology (GO) terms related to DNA binding and protein function were enriched in CD4 and CD8 memory T cells (Supplementary Figure S2). Since circulating CD8 TEM cells were upregulated in hypertension patients, they demonstrated more biological functions than CD4 TEM cells, along with rapid effector functions (Jain et al., 2018). Thus, we focused on the CD8 TEM cells in the following analysis.

### scRNA-seq analysis of CD8 TEM cells identified subpopulations enriched in hypertension

**FIGURE 3:**
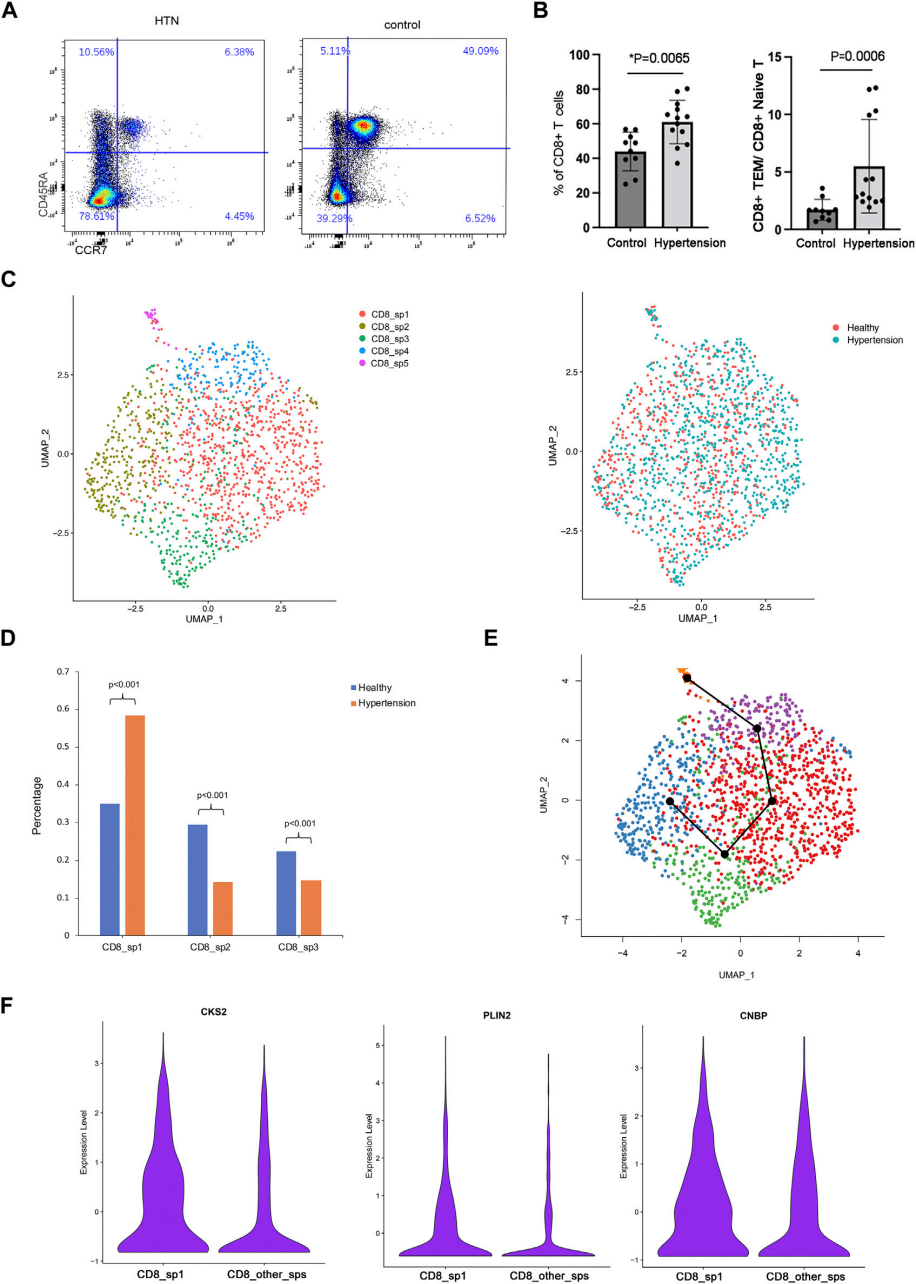
Subcluster analysis of CD8 TEM cells. **(A)** Flow cytometry analysis of CD8 effector memory T cells and the ratio of CD8 TEM to naïve T cells in the peripheral blood of healthy donors (*n* = 10) and hypertensive patients (*n* = 13). **(B)** UMAP visualization of five CD8 TEM subpopulations. **(C)** UMAP visualization of the distribution of CD8 TCM cells from healthy donors or hypertensive patients. **(E)** Trajectory reconstructed using Slingshot for CD8 TM cells. **(F)** Violin plot showing marker genes of subpopulation 1.

Human TEM cells were defined by the expression phenotype of CD45RA-CCR7-. First, we validated the frequency of CD8 TEM cells in a population of 10 healthy donors and 13 hypertensive patients (Table 2). Flow cytometry was used for this purpose, with the gating strategy shown in Supplementary Figure S1. As shown in Figure 3A, the abundance of CD8 TEM cells was markedly elevated in the peripheral blood of patients with hypertension [percentage: HTN vs. NC, 0.63 vs. 0.47, respectively; *p* = 0.0065], and the ratio of CD8 TEM cells to CD8 naïve T cells was significantly increased [percentage: HTN vs. NC, 0.038 vs. 0.016, respectively; *p* = 0.0006]. The proportion of CD8 naïve T cells was significantly decreased [percentage: HTN vs. NC, 0.17 vs. 0.30, respectively; *p* = 0.0015], while the proportion of CD8 TCM cells was comparable between the peripheral blood of normotensive controls and patients with hypertension [percentage: HTN vs. NC, 0.037 vs. 0.034, respectively; *p* = 0.52] (Supplementary Figure S3).

**TABLE 2:** Basic characteristics of normotensive controls and hypertensive patients for flow cytometry.

	CTN (n = 10)	HTN (n = 13)	*p*-value
Age, y	48.80 ± 4.44	50.77 ± 4.13	0.285
Smoke, n (%)	0	0	
Men, n (%)	60.0	61.5	1.000
SBP, mmHg	115.80 ± 7.63	149.00 ± 10.15	<0.001
DBP, mmHg	76.6 ± 6.52	93.62 ± 7.94	<0.001
BMI, kg/m^2^	25.03 ± 2.90	28.63 ± 3.75	0.02

SBP, systolic blood pressure; DBP, diastolic blood pressure; TG, triglyceride; HDL-C, high-density lipoprotein cholesterol; LDL-C, low-density lipoprotein cholesterol; FBS, fasting blood sugar; ALT, alanine transaminase; AST, aspartate transferase.

*p*-values were calculated using Student’s t-test.

To explore the role of CD8 TEM cells in hypertension, scRNA-seq analysis was further carried out. A total of five distinct subpopulations (CD8_sp1 to CD8_sp5) were identified (Figure 3B). Compared with healthy donors, patients with hypertension had a markedly elevated abundance of subpopulation 1 [percentage: HTN vs. NC, 0.58 vs. 0.35, respectively; *p* = 2.2*10^−16^] in the peripheral blood (Figures 3C,D), while subpopulation 2 [percentage: HTN vs. NC, 0.14 vs. 0.29, respectively; *p* = 0] and subpopulation 3 [percentage: HTN vs. NC, 0.15 vs. 0.22, respectively; *p* = 0.000219] were markedly decreased. Trajectory analysis inferred the differentiation trajectory of subpopulation 1 (Figure 3E). Therefore, we hypothesized that subpopulation 1, the subset with the greatest increase, may play a vital role in the development of hypertension. Further analysis was performed on this subcluster. Compared with other subclusters, subpopulation 1 differentially expressed the genes encoding cyclin-dependent kinase regulatory subunit 2 (CKS2), adipose differentiation-related protein (PLIN2), and cellular nucleic acid-binding protein (CNBP); specifically, these markers were enriched in the CD8 TEM subpopulation (Figure 3F).

### Validation of the marker genes

**FIGURE 4:**
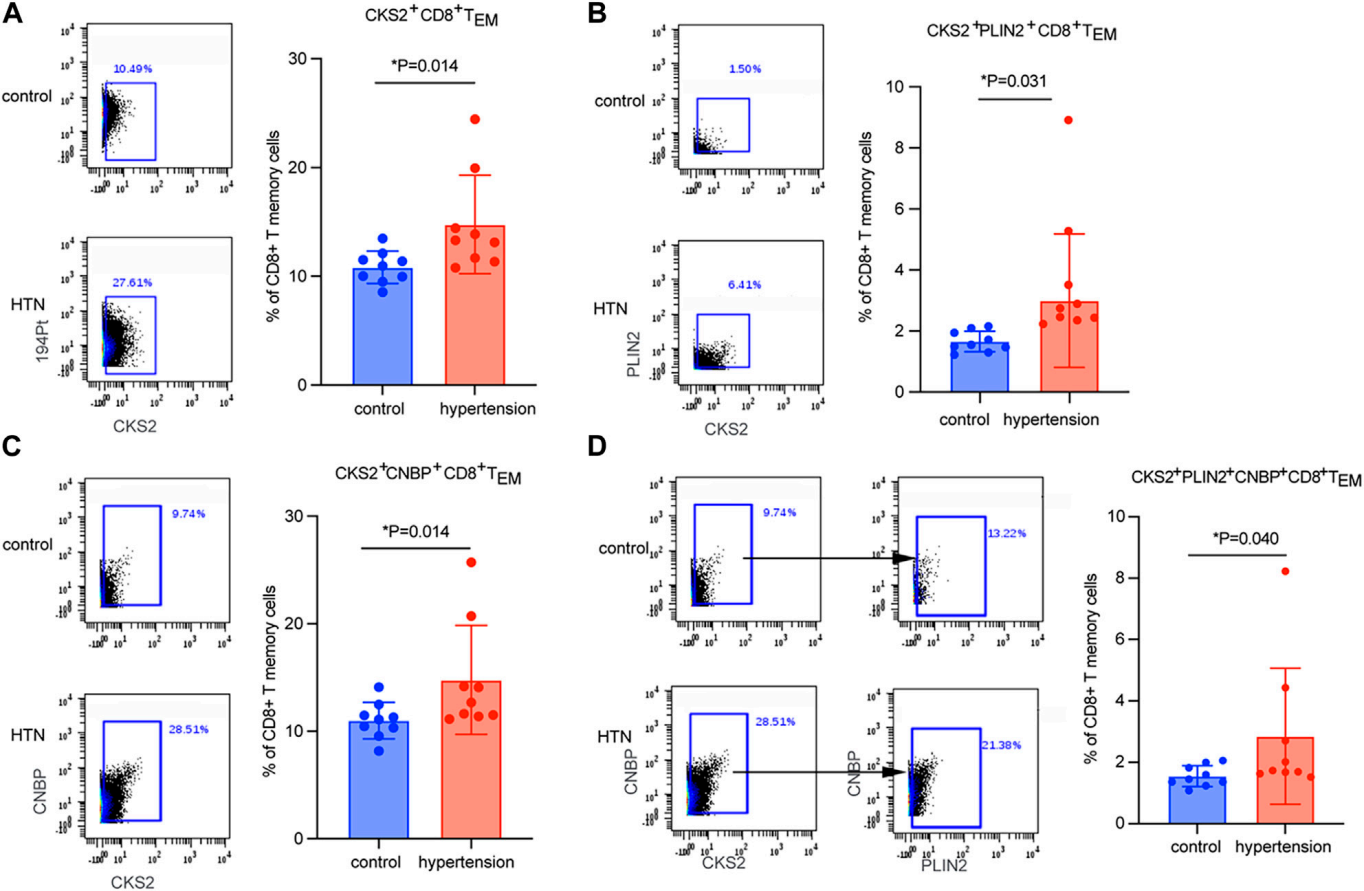
Quantitation of CD8 TEM subsets by CyTOF mass-spectrum flow cytometry. Quantitation of the percentage of **(A)** CKS2^+^ CD8 TEM cells, **(B)** CKS2^+^PLIN2^+^ CD8 TEM cells, **(C)** CKS2^+^CNBP^+^ CD8 TEM cells, and **(D)** CKS2^+^PLIN2^+^CNBP^+^ CD8 TEM cells between hypertensive patients (n = 9) and age-matched normotensive controls (n = 9).

CyTOF mass cytometry was used to measure the expression levels of key markers in cluster 1. A total of nine hypertensive patients and their age-matched controls were included (Table 3).CKS2 was proved to be the key marker gene, being differentially expressed in CD8 TEM cells between hypertensive patients and healthy controls (Figure 4A). Moreover, CKS2 CD8 TEM cells expressing PLIN2 and/or CNBP were also significantly increased in the peripheral blood of hypertensive patients (Figures 4B–D), indicating that the expression changes of these genes in CD8 TEM cells might be involved in blood pressure elevation.

**TABLE 3:** Basic characteristics of normotensive controls and hypertensive patients for CyTOF mass cytometry.

	CTN (n = 9)	HTN (n = 9)	*p*-value
Age, y	48.00 ± 5.87	50.56 ± 3.43	0.276
Smoke, n (%)	0	0	
Men, n (%)	100.0	100.0	1.000
SBP, mmHg	117.57 ± 7.52	157.89 ± 15.55	<0.001
DBP, mmHg	74.67 ± 4.30	97.56 ± 9.10	<0.001
BMI, kg/m^2^	25.40 ± 2.97	27.05 ± 2.09	0.191

SBP, systolic blood pressure; DBP, diastolic blood pressure; TG, triglyceride; HDL-C, high-density lipoprotein cholesterol; LDL-C, low-density lipoprotein cholesterol; FBS, fasting blood sugar; ALT, alanine transaminase; AST, aspartate transferase.

*p*-values were calculated using Student’s t-test.

## Discussion

Hypertensive stimuli, such as sleep apnea, repeated episodes of dietary indiscretion, or emotional stress, are intermittent and reoccurring. Recently, animal models with repeated hypertensive stimulation have been established to test whether “memory” exists in the pathogenesis of hypertension. It was reported that prior subpressor doses of angiotensin II (Ang II) treatment could sensitize animals to subsequent Ang II-induced hypertension, and this sensitization was associated with altered expression of renin–angiotensin–aldosterone system (RAAS) components in forebrain cardiovascular control structures (Xue et al., 2012). Apart from this effect on the central nervous system, the “memory” of hypertension also includes immunological memory (Itani and Harrison, 2015). Memory T cells are a group of immune cells that result from a dynamic repository of antigens and clonal expansion. These cells provide immediate protection for peripheral tissues and accumulate over the lifetime of the individuals. A previous study reported that memory T cells accumulated in the kidneys and bone marrow of mice with hypertension induced by repeated challenges with high salt or Ang II (Itani et al., 2016a; Itani et al., 2016b). In hypertensive humans, an increase in the percentage of circulating memory CD4 T and CD8 T cells was identified. These findings imply that memory T cells might have pathophysiological significance for sustaining hypertension in humans. In our study, we focused on the circulating memory T cells of hypertensive patients. By using single-cell RNA sequencing technology, subsets of memory T cells were identified.

Circulating memory T cells are a distinctive population. On the basis of distinct homing capacity and effector functions, memory T cells can be divided into TCM cells and TEM cells. TEM cells migrate to inflamed peripheral tissues and display immediate effector functions, whereas TCM cells inhabit secondary lymphoid organs and have little or no effector functions, proliferating and differentiating into effector cells in response to antigenic stimulation. In mice, TCM and TEM cells do not necessarily represent distinct subsets (Seder and Ahmed, 2003); however, in humans, these two subsets are persistent and stable (Baron et al., 2003). Our scRNA-seq results revealed TCM and TEM subtypes in hypertensive patients (Figure 1B). Based on CD8 and CD4 expression, these cells could be further subdivided into CD8 TEM, CD8 TCM, CD4 TEM, and CD4 TCM cells. It has been reported that the proportions of TCM and TEM cells in the blood vary between CD4 and CD8 cells. TCM is the predominant subtype among CD4 T cells, and the TEM subtype accounts for most CD8 T cells. Our study demonstrated similar results (Figure 2A). In addition, CD8 TCM cells are extremely rare in human peripheral blood. Within the tissues, however, TCM and TEM cells show characteristic patterns of distributions.

The underlying mechanism of essential hypertension is complex and is associated with the complex molecular network of vascular metabolism, endothelial dysfunction, inflammation, and the RAAS (Dharmashankar and Widlansky, 2010; Singh et al., 2014). The immune system plays a prominent role in the initiation and maintenance of hypertension (Singh et al., 2014). It has been noted that an excessive inflammatory response is characterized not only by an elevation of inflammatory cytokines but also by an increase in mitochondrial dysfunction, reactive oxygen species (ROS), and nitric oxide (NO). Our results regarding DEGs and functional pathways demonstrated the involvement of activated mitochondrial oxidative metabolism, vascular endothelium, and immune inflammation in circulating CD8 memory T cells, especially CD8 TEM cells (Figures 2B,C). It has been reported that TEM cells play a crucial role in blood pressure elevation and renal dysfunction that results from repeated hypertensive stimuli, and these cells are primarily responsible for the production of IFN-γ and IL-17A (Itani et al., 2016b; Xiao et al., 2020).

By taking advantage of scRNA-seq technology, which can unravel the heterogeneity and complexity of RNA transcripts within individual cells, we further analyzed CD8 TEM cells. Subpopulation 1 was the subset that was markedly elevated in the peripheral blood of patients with hypertension (Figures 3B,D). Key marker genes, including CKS2, PLIN2, and CNBP, were identified in this crucial subpopulation (Figure 3F). CKS2 is highly correlated with immunity, proliferation, and invasion (Moya et al., 2012), and high expression of this marker is associated with adverse outcomes (Yu et al., 2022). PLIN2 intrinsically acts as a perilipin protein, and its expression and phosphorylation are involved in lipolysis, lipid droplet fusion, and signal transduction (Kaushik and Cuervo, 2015; Kaushik and Cuervo, 2016). Elevated serum PLIN2 levels have been observed in response to various inflammatory stimuli, including the production of ROS and inflammatory cytokines (Marschallinger et al., 2020; Kurt et al., 2021; Zhang et al., 2021). CNBP has been identified as a regulator of NF-kB-dependent proinflammatory cytokines. It resides in the cytosol and translocates to the nucleus to activate the innate immune response, for example, enabling IL-6 gene expression, IL-12b gene transcription, and Th1 immunity (Lee et al., 2017; Chen et al., 2018). The levels of these three markers were validated by mass cytometry analysis in a new population. Mass cytometry is a variation of flow cytometry using antibodies labeled with heavy metal ion tags. Mass cytometry allows more antibodies to be combined in a single sample without significant spillover between channels. The results showed that CKS2 was the key marker gene (Figure 4A), while PLIN2^+^ CD8 TEM and CNBP^+^ CD8 TEM cells showed no significant difference in abundance between hypertensive patients and healthy controls (data not shown). However, CKS2^+^ CD8 TEM cells expressing PLIN2^+^ and/or CNBP^+^ were significantly increased in the peripheral blood of hypertensive patients (Figures 4B–D).

In conclusion, our work identified four subsets of memory T cells in the blood of hypertensive patients, with CD8 TEM cells being more numerous and demonstrating more biological functions than CD4 TEM cells. CD8 TEM cells were further analyzed using scRNA-seq technology, and subpopulation 1 was markedly elevated in individuals with high blood pressure. The key marker genes CKS2, PLIN2, and CNBP were identified and validated by mass cytometry.

## Data Availability

The datasets presented in this study can be found in online repositories. The names of the repository/repositories and accession number(s) can be found at: https://figshare.com/, 21762584, https://doi.org/10.6084/m9.figshare.21762584.v1.
