# Supplementary material for: Single-cell transcriptome profiling reveals enriched memory T-cell subpopulations in hypertension
Source: Front Cell Dev Biol. 2023 Mar 16;11:1132040. doi: 10.3389/fcell.2023.1132040 (PMC10060952; doi:10.3389/fcell.2023.1132040)
Supplement: Supplementary file 1 [file DataSheet1.PDF]

SUPPLEMENTARY INFORMATION

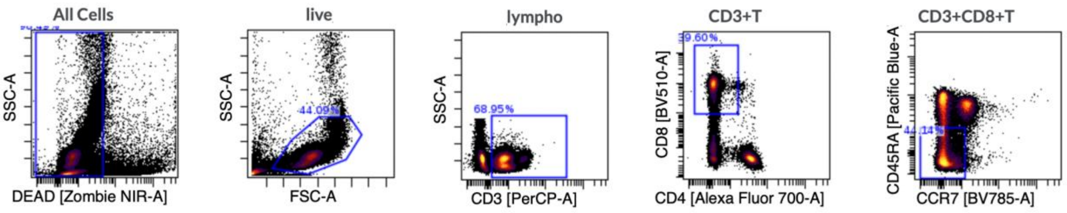

Supplementary Figure 1. Gating strategies for flow cytometry.

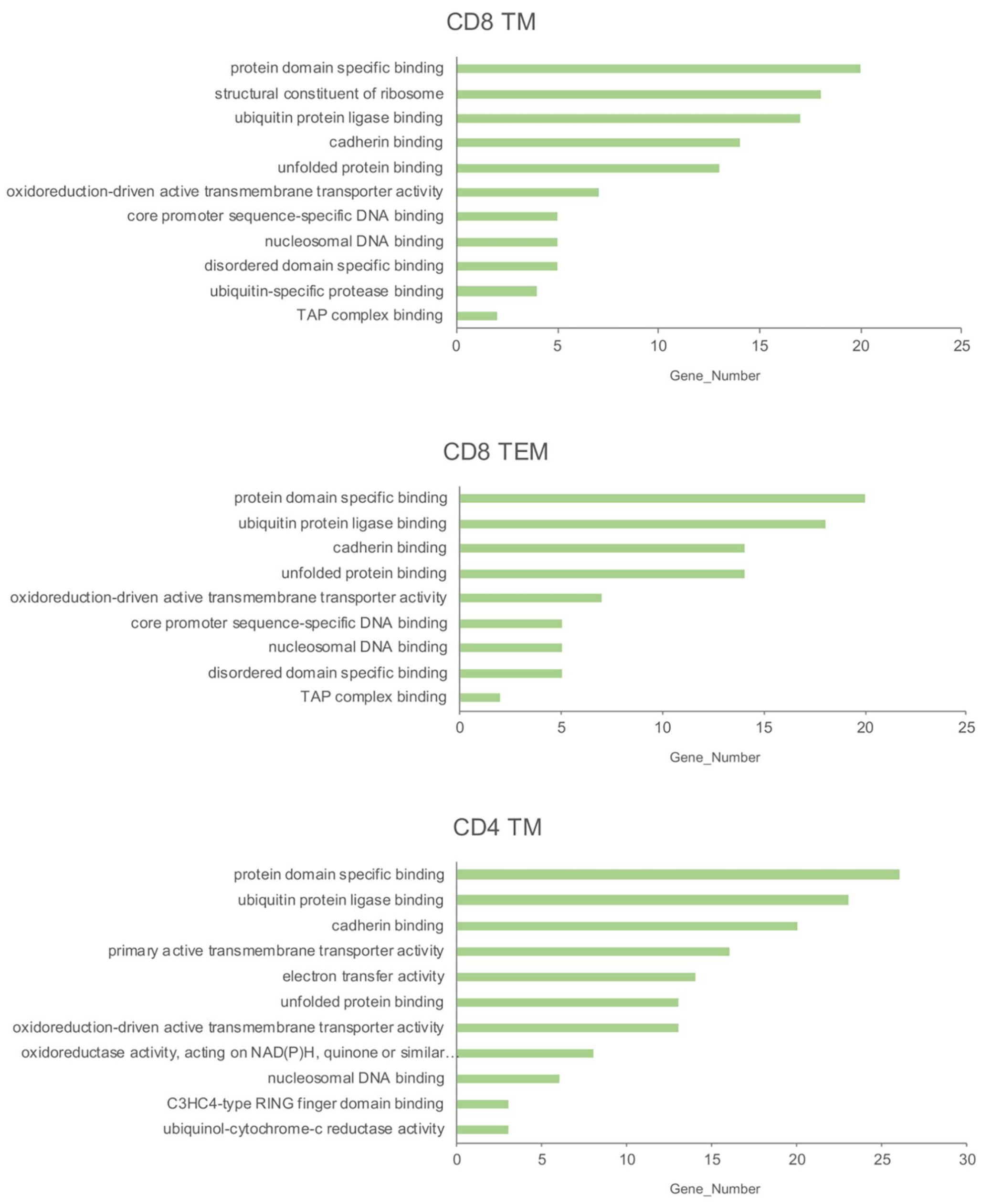

Supplementary Figure 2. **The top GO terms of differentially expressed genes (DEGs) in CD8 T cell, CD8 TEM cells and CD4 TM cells.**

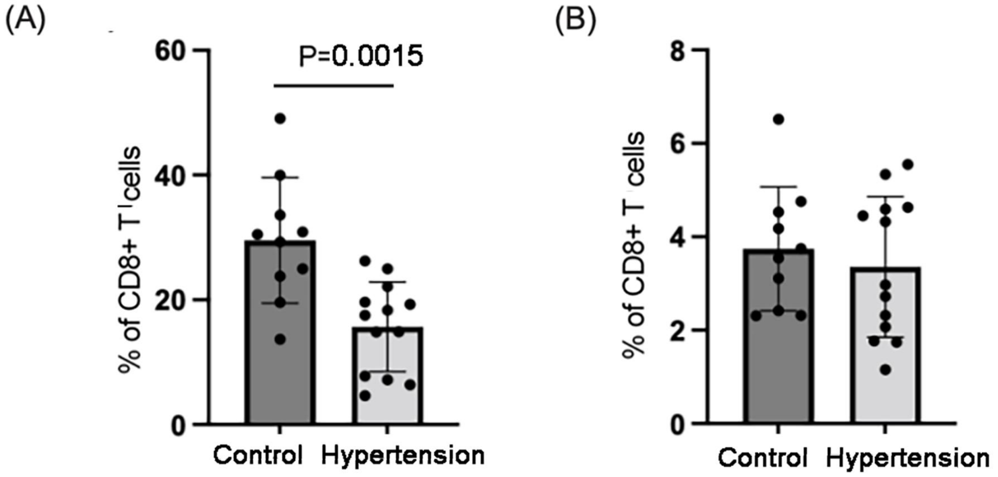

Supplementary Figure 3. **Frequency of CD8+ naïve T cells and CD8+ central memory T cells in the peripheral blood of hypertensive patients and their healthy controls.** (A) Frequencies of CD8 naïve T cells ($p = 0.0015$). (B) Frequencies of CD8 central memory T cells ($p = 0.52$). Lines and error bars are presented as mean ± SEM.

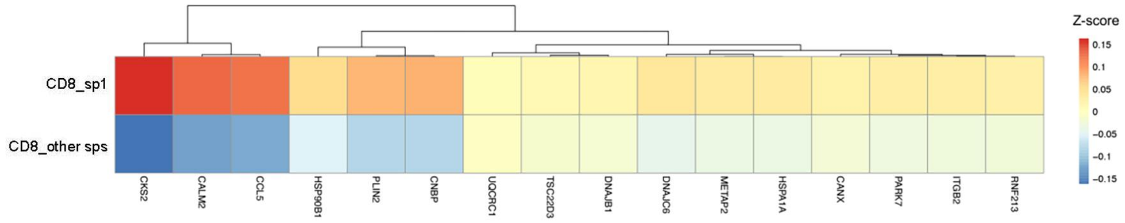

Supplementary Figure 4. Heatmapshowing marker genes of subpopulation 1.

Supplementary Table 1.Antibodies used in this research.

Reagent	Company	Catalog	Clone
Zombie NIR	Biolegend	423105	-
Percp anti-human CD3	Biolegend	300427	UCHT1
Alexa Fluor 700 anti-human CD4	Biolegend	357417	A161A1
BV510 anti-human CD8a	Biolegend	301047	RPA-T8
Pacific Blue anti-human CD45RA	Biolegend	304118	HI100
BV785 anti-human CCR7	Biolegend	353230	G043H7

Supplementary Table 2. Basic statistics of scRNA-seq data sets.

Sample ID	Total reads pairs	Total mapped reads	Gene counts	Cell counts	UMI counts
N1	201203544	114882252	39870	508	27565301
N2	228231119	128628738	39870	539	31610840
N3	135420105	98220756	35416	207	19704348
N4	176836304	130043839	35416	312	25128676
N5	568172533	453174869	40605	735	69033744
H1	241640481	164243757	42746	720	33588944
H2	263597804	182435910	42746	718	35409341
H3	481378537	368011798	44099	422	86862723
H4	155596478	120259193	40605	699	19616984
H5	421150031	317250196	44099	557	77961834
